# Print‐and‐Grow within a Novel Support Material for 3D Bioprinting and Post‐Printing Tissue Growth

**DOI:** 10.1002/advs.202200882

**Published:** 2022-10-19

**Authors:** Majd Machour, Noy Hen, Idit Goldfracht, Dina Safina, Maya Davidovich‐Pinhas, Havazelet Bianco‐Peled, Shulamit Levenberg

**Affiliations:** ^1^ Department of Biomedical Engineering Technion – Israel Institute of Technology Haifa 32000 Israel; ^2^ Department of Chemical Engineering Technion – Israel Institute of Technology Haifa 32000 Israel; ^3^ The Norman Seiden Multidisciplinary Program for Nanoscience and Nanotechnology Technion – Israel Institute of Technology Haifa 32000 Israel; ^4^ Department of Biotechnology and Food Engineering Technion – Israel Institute of Technology Haifa 32000 Israel

**Keywords:** 3D bioprinting, bioink, contraction, *κ*‐Carrageenan, support material, tissue engineering

## Abstract

3D bioprinting holds great promise for tissue engineering, with extrusion bioprinting in suspended hydrogels becoming the leading bioprinting technique in recent years. In this method, living cells are incorporated within bioinks, extruded layer by layer into a granular support material followed by gelation of the bioink through diverse cross‐linking mechanisms. This approach offers high fidelity and precise fabrication of complex structures mimicking living tissue properties. However, the transition of cell mass mixed with the bioink into functional native‐like tissue requires post‐printing cultivation in vitro. An often‐overlooked drawback of 3D bioprinting is the nonuniform shrinkage and deformation of printed constructs during the post‐printing tissue maturation period, leading to highly variable engineered constructs with unpredictable size and shape. This limitation poses a challenge for the technology to meet applicative requirements. A novel technology of “print‐and‐grow,” involving 3D bioprinting and subsequent cultivation in *κ*‐Carrageenan‐based microgels (CarGrow) for days is presented. CarGrow enhances the long‐term structural stability of the printed objects by providing mechanical support. Moreover, this technology provides a possibility for live imaging to monitor tissue maturation. The “print‐and‐grow” method demonstrates accurate bioprinting with high tissue viability and functionality while preserving the construct's shape and size.

## Graphical abstract

A new printing support material (CarGrow) composed of *κ*‐Carrageenan microgels is developed for the long‐term cultivation of 3D printing cellular scaffolds. The cultivation of cellular constructs within CarGrow provides a favorable cell environment that minimizes the contraction phenomena of low viscosity hydrogels combined with cells while allowing for live‐cell imaging.

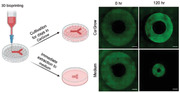

## 1 Introduction

A major challenge in tissue engineering is fabricating 3D constructs with structural and functional properties that mimic the 3D environment of living tissue in a reproducible and stable manner.^[^ ^1^ ^]^ Recently, 3D bioprinting has gained broad interest for its potential to create such constructs. Specifically, extrusion‐based bioprinting emerged as a valuable technique to fabricate cell‐containing 3D scaffolds with high complexity and accuracy.^[^ ^2^ ^]^ In this technique, a wide range of soft biomaterials with physiological‐like properties termed bioinks can be incorporated with living cells and extruded through a nozzle in a layer‐by‐layer manner. Following extrusion, the bioinks undergo gelation through diverse cross‐linking mechanisms, either during or after the printing process.^[^ ^3^ ^]^

An inherent limitation of extrusion printing onto a hard surface is the deformation and collapse of the extruded soft bioinks.^[^ ^4^, ^5^ ^]^ Utilization of high viscosity bioinks can prevent this issue; however, increasing the viscosity can interfere with the cellular functionality. The method of freeform direct printing into a granular support material was developed to overcome this obstacle.^[^ ^6^, ^7^ ^]^ Support materials are yield stress fluids with shear‐thinning and self‐healing properties, composed of suspended particles. They liquefy under high shear stress but behave as a rigid matrix under low shear stress.^[^ ^8^, ^9^ ^]^ Thus, the support material allows for free movement of the nozzle needle during printing while maintaining the structure by trapping the extruded bioink between the particles upon removing the stress. Next, the printed structure is cured, followed by removing the support material using an external trigger such as temperature change,^[^ ^10^ ^]^ enzymatic cleavage,^[^ ^11^ ^]^ or mechanical force.^[^ ^12^, ^13^ ^]^ Finally, the printed construct is transferred into a liquid medium for tissue maturation and growth. Direct printing within support materials can provide mechanical support to low viscosity biomaterials, which are compatible with living cells.^[^ ^14^ ^]^ This technique was successfully applied while utilizing various types of support materials, including polyacrylic acid microgels,^[^ ^7^, ^15^ ^]^ gelatin microparticles,^[^ ^6^, ^10^ ^]^ modified hyaluronic acid,^[^ ^16^ ^]^ agarose fluid gel,^[^ ^12^ ^]^ agar microparticles,^[^ ^17^ ^]^ nano clay,^[^ ^18^, ^19^ ^]^ alginate microgels,^[^ ^20^ ^]^ gellan fluid gel,^[^ ^13^ ^]^ and alginate–xanthan gum hybrid.^[^ ^11^ ^]^ This bioprinting technique achieves high fidelity and allows for the precise fabrication of complex structures based on the living tissue and organ properties.

Most researchers focus on printability and immediate bioprinting outcomes. However, the transition from cells mixed in a hydrogel into a functional tissue requires further cultivation post‐printing. During this period, the printed cells differentiate, secrete extracellular matrix (ECM) components, and establish intercellular connections.^[^ ^21^ ^]^ Engineered tissue maturation prior to implantation is an integral part of various tissue engineering approaches,^[^ ^22^, ^23^ ^]^ since it allows for differentiation of stem cells into desired tissue types,^[^ ^24^ ^]^ maturation of muscle fibers,^[^ ^25^ ^]^ ossification of bone tissue,^[^ ^26^ ^]^ and establishment of a mature self‐assembled microcapillary network that enhances implant survival and integration within the host tissue.^[^^]^ Therefore, there is a need to preserve the stability of printed tissues during cultivation for tissue repair or disease modeling applications. Nevertheless, in many cases, the shape, size, and structural complexity of the cell‐containing printed construct cannot be maintained over time due to the contraction of the hydrogel. The instability results from contractile forces applied by the cells on the bioink matrix. Due to insufficient strength, the hydrogel matrix buckles, leading to substantial shrinkage and deformation, especially for high cell density constructs.^[^ ^29^ ^]^ Hydrogel deformation reduces the volume of the construct and presents several difficulties. First, for tissue replacement applications, the design of the construct is based on patient‐specific imaging, which the implanted engineered tissue needs to accommodate. Cellular construct contraction disrupts the structure hence presenting a significant hurdle for tissue implantation. Second, the volume reduction leads to increased cell density, a denser network, and reduced porosity within the construct. Since these variables play an essential role in cell viability, differentiation state, and function,^[^ ^30^, ^31^ ^]^ the contraction of the hydrogel might lead to undesired effects on the engineered tissue. Furthermore, the degree of shrinkage might vary depending on cell type, cell passage, and variability between cells or hydrogel batches. We note that many studies describe only the hydrogel's initial geometry and cell density without reporting on the final dimensions of the construct. This lack of information makes reproducing the same results by similar experiments complex and unpredictable.

A few methods were suggested to limit hydrogel contraction.^[^ ^32^ ^]^ For example, printed supporting structures made from a rigid synthetic material such as polycaprolactone were used to encapsulate cell/bioink mixture.^[^ ^33^ ^]^ However, these supporting structures prolonged the in vivo degradation time. Moreover, this method often requires high‐temperature extrusion printing of the polymer ink in proximity to cell‐laden hydrogels, a fabrication process that might reduce cell viability. Thus, a facile method of counteracting contraction of bioprinted constructs is needed, where the support is external and removable, with minimal effect on the cell's viability and function.

Here, we present a novel approach in 3D bioprinting into a granular support material aimed to enhance the long‐term structural stability of the printed objects. In this new “print‐and‐grow” approach, the printed hydrogel scaffold is not removed from the support material immediately after printing but instead cultivated within it for prolonged periods (days to weeks). Previous works have demonstrated the viability and motility of cells that are cultivated for days in granular support materials,^[^ ^34^, ^35^, ^36^ ^]^ however, the cells were mixed with medium without any biomaterials to generate a cellular scaffold. To our knowledge, this work is the first to describe the viability, functionality, and stability of cellular hydrogel scaffolds for tissue replacements after a prolonged culture period within support materials. We hypothesized that bioprinting followed by tissue maturation within the same support material might reduce post‐printing contraction and deformation. Therefore, we anticipated it would enable printing complex structures using low viscosity biomaterials while providing a cell‐friendly environment. A second envisioned advantage of the “print‐and‐grow” method is allowing for direct live‐cell imaging during culture without the need to extract the construct from the granular hydrogel. This work describes the fabrication of new granular material based on *κ*‐Carrageenan microgels (CarGrow) suitable for the “print‐and‐grow” approach. *κ*‐Carrageenan is a biocompatible polysaccharide originating from red seaweeds and is widely used in the food industry as a thickening agent.^[^ ^37^ ^]^ Moreover, *κ*‐Carrageenan is a thermosensitive polymer that undergoes gelation during cooling or in the presence of certain cations. The gelation mechanism involves coil to helix transition during cooling and, if salts are present, ionic interaction of the sulfate groups with cations. These interactions inhibit electrostatic repulsion between the helix chains and promote secondary aggregation to form a transparent hydrogel.^[^ ^38^ ^]^ Previous studies have shown that potassium chloride (KCl) generates a resilient macroscopic hydrogel;^[^ ^39^ ^]^ thus, we exploit this property to fabricate stable microgels without using any toxic cross‐linking agents, as described below. The properties of CarGrow support material allow for 3D bioprinting of various bioinks, enable prolonged cultivation of viable and functional bioprinted tissue, and substantially reduced bioprinted construct contraction. Additionally, the transparency of CarGrow provides the possibility for direct live‐cell imaging during culture without the need to extract the construct from the granular hydrogel.

## 2 Results and Discussion

### 2.1 Preparation and Characterization of CarGrow Microgels

The main design criteria for a support material suitable for the print‐and‐grow approach are rheological compatibility with freeform 3D bioprinting and stability under culturing conditions for prolonged times. Obviously, biocompatibility and lack of cytotoxicity are also required. Other key factors include optical clarity that enables live‐cell imaging, and the possibility of controllable fabrication. Although previous studies have used other granular hydrogels as support materials, they hold significant limitations for long‐term culture application including temperature sensitivity,^[^ ^10^ ^]^ ions sensitivity that can lead to precipitation,^[^ ^7^, ^40^ ^]^ or transparency.^[^ ^12^ ^]^ Based on these considerations, we chose to design and investigate a new support material composed of *κ*‐Carrageenan microgels (CarGrow) fabricated using water in oil (W/O) emulsion. Microgels are polymeric microscale particles with high water content and porosity.^[^ ^41^ ^]^ Since microgels can sediment into a microporous granular hydrogel and flow under high shear stress, we hypothesized that they provide shear‐thinning and self‐healing properties appropriate for printing.

**Figure 1:**
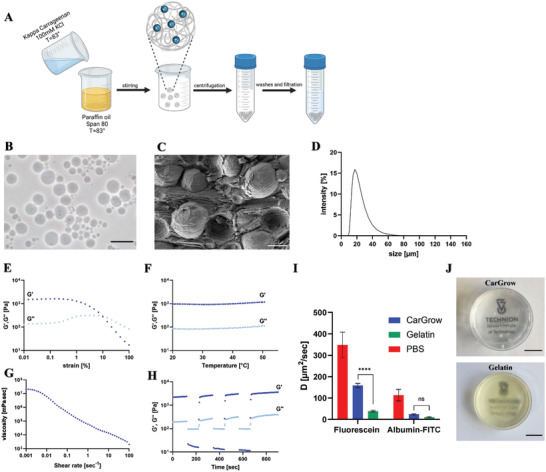
CarGrow characterization. A) The fabrication process of the microgels: W/O emulsion is used to fabricate spherical microgels from *κ*‐Carrageenan. The water droplets in the continuous phase are formed using mechanical stirring and are stabilized using the nonionic surfactant Span 80. During cooling, the polymer chains in the water droplets undergo ionic gelation and generate spherical microgels. The formed microgels are separate from the oil phase using centrifugation. B) Light microscopy image of dilute microgels (scale bar: 50 µm) and C) cryo‐SEM image of the sedimented microgels within the continuous phase (0.1m KCl) (scale bar: 20 µm). The images demonstrate the spherical morphology of the particles. D) Size distribution of the microgels (*n* = 6). Rheological characterization of the microgels using E) strain sweep test, F) temperature sweep test, G) steady shear flow test, and H) thixotropic test (*n* = 3, *G'*: storage modulus dark blue, *G''*: Loss modulus light blue). I) The diffusion coefficient of fluorescein tracer (376 Da) and Albumin‐FITC (66.4 kDa) through PBS (red), CarGrow (blue), and gelatin support material (green) was calculated using FRAP experiment (*n* ≥ 3). J) Photographs of CarGrow and gelatin support bath placed on the university logo photograph demonstrate their transparency (scale bar: 10 µm). ns: not significant, ****p < 0.0001.

Our first goal was to fabricate spherical particles in a facile and reproducible manner. Hence, we developed a method based on first creating a W/O emulsion at high temperature, then cooling the emulsion to induce gelation, and finally separating the microgels (**Figure** **1**A). More specifically, an aqueous precursor solution containing *κ*‐Carrageenan and ionic cross‐linker (KCl) was mixed with an oil phase at a high temperature in which the polymer is soluble and stirred to generate water droplets within the continuous oil phase. The polymeric water droplets were stabilized using Span 80 as a surfactant. The hydrogels cure and solidify upon cooling to room temperature, which allows their separation using centrifugation. The spherical morphology of the obtained microgels is visible in bright field microscopy images (Figure 1B) and cryogenic scanning electron microscopy (cryo‐SEM) micrographs (Figure 1C). The size distribution of the microgels indicated that small particles with an average diameter of 21.4 ± 2.1 µm were obtained (Figure 1D).

It is well known that the rheological properties of a successful support material should include shear‐thinning and self‐healing behaviors.^[^ ^14^ ^]^ Hence, we aimed to measure these critical rheological behaviors of CarGrow. First, we performed a strain sweep experiment to explore the microgels suspension's linear viscoelastic region (LVR, Figure 1E). The material viscoelastic properties can be expressed using the storage (*G'*) and loss (*G''*) moduli. The results demonstrate the transition from “solid like” behavior characterized with *G'* > *G''* to “liquid like” behavior characterized with *G''* > *G'* due to high shear strains. Next, we performed temperature ramp experiments to evaluate changes in the *G'* and *G''* in response to temperature increase (Figure 1F). The results revealed a solid gel‐like behavior characterized with *G'* > *G''* in the entire tested range, including ambient and physiological temperatures. This behavior should enable constant mechanical support of the solid particles during and post‐printing. The shear‐thinning behavior was studied using flow measurement (Figure 1G) to assess the viscosity at increasing shear rates. This experiment simulates the needle movement through the support material. The microgels bed exhibited a decrease in viscosity with an increase of shear rate, which was well fitted to a simple power‐law model^[^ ^42^ ^]^

(1) $$\eta =k{\dot{\gamma }}^{n-1}$$

where *η* is the measured viscosity, $\dot{\gamma }$ is the shear rate, *k* is the consistency coefficient, and *n* is the power‐law index or flow behavior index. The power‐law index depends on the shear‐thinning characteristics; *n* < 1 for shear‐thinning fluids whereas *n* > 1 for shear thickening materials. As expected from the qualitative observation showing that the viscosity decreases with shear rate, the calculated flow index (*n* = 0.21 ± 0.01) suggests a shear‐thinning behavior of the microgels support. Finally, thixotropy measurements were performed to characterize the self‐healing and recovery properties. These experiments apply cycles of small amplitude oscillations (*γ* = 0.1%) to test the initial microgels bed properties. Then, large amplitude oscillations (*γ* = 100%) were applied to disrupt the structure of the packed microgels and evaluate the recovery to the initial properties (Figure 1H). In general, the results exhibited gel behavior under low shear strains characterized with *G'* > *G''* and a liquid behavior under high shear strains characterized with *G''* > *G'*. More importantly, rapid and complete recovery was demonstrated in all cycles indicating the self‐healing properties of CarGrow. These fundamental rheological properties are similar to those of previously studied granular materials composed of polysaccharides particles^[^ ^12^, ^13^, ^20^ ^]^ and emphasize the potential to use CarGrow as support material for bioprinting.

In order to grow tissue constructs within the support material, it is necessary to ensure nutrient diffusion.^[^ ^43^ ^]^ Therefore, we sought to test the diffusion of a small and large molecule through the support material using fluorescence recovery after photobleaching (FRAP). The diffusion coefficient of fluorescein tracer (376 Da) and Albumin‐FITC (66.4 kDa) through CarGrow was calculated and compared to the diffusion coefficients of the same molecule in gelatin support bath (LifeSupport) and 1X phosphate buffer (PBS, Figure 1I). As expected, the diffusion coefficients in the granular gel beds were smaller than the values measured in a liquid buffer. Furthermore, the diffusion coefficient through CarGrow was higher than in the gelatin support bath for the fluorescein tracer, however no significant difference was observed for the large molecule Albumin‐FITC. Despite this, a significant difference was found in the immobile fraction that was observed in the bleach area of LifeSupport for Albumin‐FITC tracer (Figure S1A, Supporting Information). 71.3% of immobile fraction was calculated for the gelatin particles while 4.9% was found for the CarGrow microgels. This significant difference in the immobile fraction of Albumin‐FITC between CarGrow and LifeSupport emphasizes the potential for better protein transport within CarGrow and might be attributed to protein–protein interactions within LifeSupport. To visualize the difference in the immobile fraction, we performed diffusion test within a single particle using FRAP. Figure S1B in the Supporting Information demonstrates full recovery of the fluorescent Albumin‐FITC within CarGrow compared to the gelatin particle. These findings of an immobile fraction do not align with previous works on macroscopic gelatin hydrogels and therefore raise questions about the influence of the spherical morphology, gelatin concentration, and the impact of additional materials on the diffusion of fluorescent tracers.^[^ ^44^, ^45^ ^]^ Microgels can be used as a soft matrix for tissue maturation owning to the high‐water content that provides a cell‐friendly environment alongside mechanical support to the printed structure. Previous work demonstrates the potential to grow cells in a soft hydrogel medium composed of synthetic microgels.^[^ ^34^ ^]^ However, the research focused on cell viability, motion, and proliferation rather than the generation of cellular scaffolds and investigation of tissue maturation and contraction. Our results demonstrate that molecular transport is possible through the void spaces between the microgels and further prove the potential to grow cellular constructs within CarGrow.

In addition to the morphology, porosity, and rheological properties, an essential feature of CarGrow is its transparency. The printing process involves multiple parameters that affect the printability of low viscosity biomaterials; therefore visualization of the printed structure through the support material can ease and improve the calibration of the printer and the bioink.^[^ ^7^, ^11^, ^20^ ^]^ Moreover, the transparency of the microgels enables direct live‐cell imaging during culture without the need to extract the printed constructs. To quantify the transparency of the microgels, we measured the absorbance of light passing through CarGrow, and the transmittance spectra was calculated and compared to a turbid support material composed of gelatin particles (Figure S2, Supporting Information). The average transmittance of CarGrow was 56%, and the transmittance of the gelatin particles was 23%. Figure 1J demonstrates the visual difference between the turbid and the transparent support material.

### 2.2 CarGrow Enables High‐Fidelity Printing of Various Bioinks

**Figure 2:**
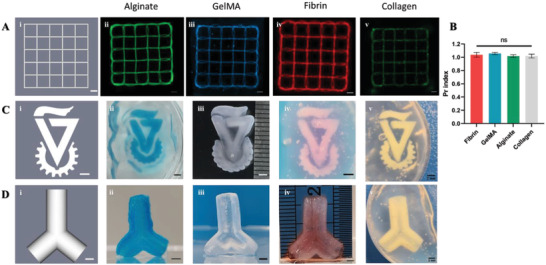
3D printing within CarGrow microgels and shape fidelity evaluation. A) i) Computer design of rectilinear pattern and representative images after printing of ii) alginate, iii) GelMA, iv) fibrin, and v) collagen bioinks within CarGrow (fluorescent microspheres were added for visualization) (scale bar: 1 mm). B) A semiquantitative evaluation of the geometric accuracy of a single internal square in the rectilinear pattern (*n* = 3). 3D printing of volumetric constructs: C) i) Computer design of the university logo and photographs after printing within CarGrow of ii) alginate with alcian blue dye, iv) fibrin, and v) collagen bioinks. iii) The GelMA structure was extracted from CarGrow (scale bar: 2 mm). D) i) Computer design of hollow diverging vessel and photographs after extraction of ii) alginate with alcian blue dye, iii) GelMA, and iv) fibrin bioinks. v) The collagen structure within CarGrow (scale bar: 2 mm). ns: not significant.

A versatile support material needs to support various types of extruded bioinks with different gelation mechanisms.^[^ ^8^, ^46^ ^]^ We tested four widely used bioinks: alginate with calcium chloride as ionic cross‐linker, gelatin‐methacryloyl (GelMA) that undergoes photo‐cross‐linking, fibrinogen which undergoes enzymatic cross‐linking by thrombin and collagen with thermal gelation at 37 °C. To verify the bioink printability within CarGrow and quantify shape fidelity, we printed a planar structure of four layers rectilinear pattern with an internal square shape of 2 mm x 2 mm. To aid the visualization of the extruded bioinks, either alcian blue powder or 0.5 µm fluorescent microspheres were mixed with the bioink. **Figure** **2**A shows the similarity of the printed structures to the original design for all bioink types. To further examine the geometric accuracy of the printed structure within CarGrow, we performed a semiquantitative evaluation by calculating the printability index of internal square (Pr).^[^ ^47^, ^48^ ^]^ The printability index can be used to evaluate ideal gelation of bioinks which is characterized by smooth and perfect morphology of extruded filaments. When printing grid pattern, a square hole with circularity of *π*/4 demonstrates high fidelity and proper gelation condition of the bioink. The printability index is defined as the ratio between the area and the perimeter of the square hole and where it strives to 1 the circularity is *π*/4. Here, we utilized this analysis as a proof of concept to verify the geometric accuracy of bioinks within CarGrow and demonstrate high fidelity grids patterns. Figure 2B compares the calculated indexes of the printed bioinks. The lack of significant difference between the indexes emphasizes the versatility of CarGrow and its ability to support different gelation mechanisms.

Unlike planar structures, biological scaffolds are typically 3D bulk objects with complex architecture. Therefore, we aimed to print a large‐scale volumetric construct within CarGrow. First, we printed the Technion university logo using several bioinks to demonstrate the printing accuracy of complex structures (Figure 2C). To visualize the fidelity between the CAD design and the printed constructs, we printed the Technion university logo as well as a diverging vessel structure using alginate bioink and overlayed the CAD design on top of the imaged printed constructs (Figure S3A, Supporting Information). The overlap between the images emphasizes the ability to print complex structures accurately. The slight deviation from the original design can be associated with swelling or shrinkage of hydrogels after cross‐linking. Next, we printed a vascular‐like structure since blood vessels have a critical role in providing nutrition and oxygen transport in cellular scaffolds.^[^ ^43^ ^]^ A hollow diverging vessel was printed using the same bioinks and extracted from CarGrow using gentle pipetting (Figure 2D). To demonstrate the open lumen of the structure, an aqueous red food dye solution was perfused through the channels (Movie S1, Supporting Information). The hollow vessels were perfusable and robust, validating the high resolution and fidelity of the constructs (Figure S3B, Supporting Information). Moreover, the resulting structures of alginate, GelMA, and fibrin had sufficient structural integrity to be extracted from CarGrow and handled freely. However, collagen structures had insufficient structural stiffness to be handled freely in air. Low concentration collagen printing presents a major challenge due to its low viscosity, slow thermal cross‐linking, and insufficient integrity of the structure. Nevertheless, we chose to print collagen at a relatively low concentration of 5 mg mL^−1^, which is suitable for encapsulating cells, as collagen is the main component of the ECM and an excellent candidate bioink for cell printing. Our results demonstrate the advantage to use CarGrow as support material for collagen printing arising from its ability to provide prolonged mechanical support even at 37 °C.

### 2.3 Print‐and‐Grow Approach Limits the Contraction of Cell‐Laden Bioprinted Constructs

**Figure 3:**
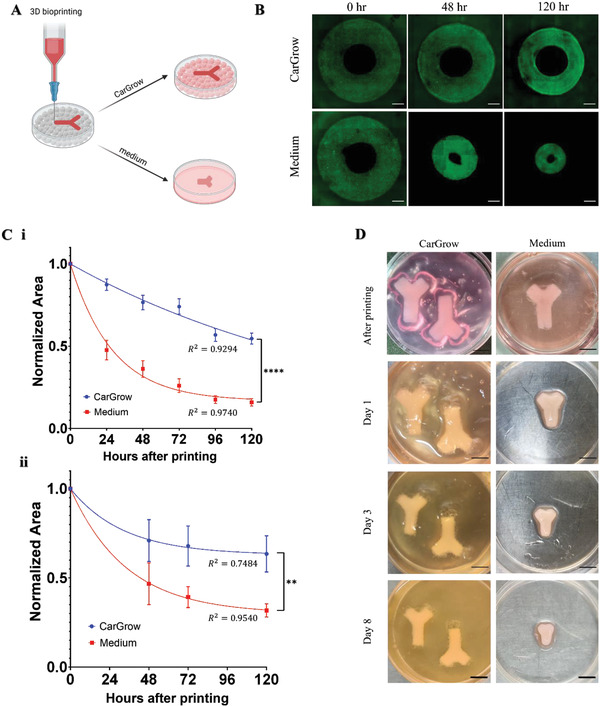
Incubation in CarGrow reduces cellular construct contraction. A) Illustration of the printing process: in the classical approach, the support material is removed and replaced with a liquid medium shortly after printing and cross‐linking. Consequently, the cellular scaffold shrinks due to cell exerted forces and the original structure deform. We suggest maturing the cellular scaffold in the support material to minimize the structure contraction. B) Fluorescence images of printed HNDF construct extracted to liquid medium or remained in CarGrow during 5 days of culture (scale bar: 1 mm). C) Quantification of the normalized printed area of i) HNDF and ii) MSC constructs during 120 h of culture. (*n* = 6). Continuous lines are the fitted exponential decay curves. D) Photographs of a hollow diverging vessel structure containing HNDF cells encapsulated in fibrin‐based bioink. The printed constructs were extracted to liquid medium (right) or cultivated in CarGrow (left) for 8 days of culture (scale bar: 5 mm). Scaffolds incubated in the medium shrunk significantly compared to scaffolds cultured within CarGrow. **p < 0.01, ****p < 0.0001.

The dimensions of clinically relevant engineered constructs should be based on patient‐specific scans. These dimensions should be maintained throughout the in vitro culture period,^[^ ^49^ ^]^ allowing for tissue maturation prior to implantation. Existing techniques of 3D printing of cell‐laden hydrogels into granular materials hamper the construct structural stability. The support materials are usually removed after bioink curing, and the construct is cultured in a liquid medium.^[^ ^6^, ^10^, ^11^, ^12^ ^]^ This transition from granular hydrogel to liquid phase significantly reduced the mechanical support provided by the granular media and promoted construct contraction during the culture period. It is important to clarify that not all the cells are equally contractile (e.g., mature adipocytes are noncontractile cells), hence not all printed cellular scaffolds will shrink. Consequently, one of the aims of this work was to investigate the contraction phenomena of contractile cells and develop a support material that allows the long‐term incubation of printed constructs. We hypothesize that a continuous process of bioprinting and subsequent tissue cultivation within the CarGrow will counteract the contraction of the tissue. To investigate the effect of incubation within CarGrow on the contraction of printed cellular scaffolds, we compared the overtime size change between cellular constructs cultivated within CarGrow and extracted from CarGrow immediately after cross‐linking. We studied the shrinkage behavior of cellular constructs bioprinted into CarGrow using a fibrinogen‐based bioink. We used either mesenchymal stem cells (MSCs) or human normal dermal fibroblasts (HNDFs); both cell types were fluorescently labeled to enable post‐printing live‐cell imaging. The constructs were printed in a concentric disk shape with an outer and inner diameter of 7 and 3 mm, respectively, and a height of 2 mm (Figure S4A, Supporting Information). Following printing, the constructs were allowed to cross‐link for 30 min at 37 °C and were subsequently either cultured within CarGrow or extracted from CarGrow by gentle pipetting and transferred to the appropriate medium (**Figure** **3**A). For MSCs containing constructs, an osteogenic medium was used to induce post‐printing differentiation of MSCs into osteoblasts. We live imaged the cells immediately after printing and then imaged them daily for 5 consecutive days (Figure 3B and Figure S4B, Supporting Information). The inner and outer diameters were measured, and the total area of the constructs was calculated and normalized to the initial area after printing (Figure 3C).

After 48 h of cultivation, the extracted constructs significantly shrunk, and their total area was reduced to 36 ± 5% of its initial value for HNDF and to 47 ± 10% of its initial value for MSCs. On the other hand, scaffolds incubated in CarGrow exhibited significantly smaller contraction, and their total area was reduced to 77 ± 4% and 71 ± 10% of its initial value for HNDFs and MSCs, respectively. Furthermore, after 5 days of cultivation, extracted HNDF constructs incubated in the liquid medium shrunk to 16 ± 2% of the original area, whereas scaffold incubated within CarGrow only shrunk to 55 ± 3% of the starting area. This significant difference was also observed after 5 days of culturing the MSCs scaffolds where the area of the extracted constructs reduced to 32 ± 3% of its original value while scaffolds incubated within CarGrow reduced to 63 ± 9% of the original area. Due to the ring geometry of the printed constructs and the presence of microgels in the center, the effect of CarGrow on size reduction was more pronounced on the printed structures’ inner diameter than the outer diameter (Figure S4C–F, Supporting Information). If we assume that the construct's contraction rate is proportional to its area, then we can describe the contraction kinetics using the following differential equation

(2) $$\frac{dA\left(t\right)}{dt}=\alpha \cdot A\left(t\right)$$

where *A*(*t*) is the instantaneous area and *α* is the rate of contraction. Solving this equation gives us an exponential decay function that describes the construct's area as a function of time^[^ ^50^ ^]^

(3) $$A\;\left(t\right)=\left(A_{i}-A_{f}\right)\cdot e^{\alpha \cdot t}+A_{f}$$

where *A*_i_ and *A*_f_ are the initial and final areas, respectively. Next, we can attempt to fit the experimental data to this equation and plot the fitted curve. The contraction kinetics of constructs incubated in medium show a relatively good fit to the exponential decay model for both MSC and HNDF constructs (Figure 3C), while the contraction kinetics of CarGrow cultivated constructs do not fit this model. The different contraction kinetics in the two culture methods are indicative of different mechanical systems. The contraction results emphasize the advantage of growing cellular scaffolds within support materials and open a new approach to print‐and‐grow cellular hydrogels. Importantly, this experiment also demonstrates that CarGrow enables direct live‐cell imaging during culture.

Next, we aimed to examine the contraction of HNDF constructs incubated within CarGrow for 5 days and then extracted to liquid medium for an additional 5 days. The constructs contracted rapidly after extraction to medium, reaching a final area similar to constructs extracted immediately to medium (Figure S4G,H, Supporting Information). HNDFs are contractile in their nature, regardless of their maturity. Therefore, it is expected that they will continue shrinking after extraction. It should be noted that this phenomenon is dependent on cell type since the contractility might change during differentiation or maturation. For instance, MSCs are highly contractile cells, that during differentiation into different lineages, lose some or all their contractility.

The contraction of printed constructs can in some instances be used advantageously to achieve certain results. For example, researchers used an anchoring type of support structures, where the contraction is limited in one direction.^[^ ^33^ ^]^ This led to a directional contraction of the structure which aided the development of aligned muscle fibers. However, this type of support is very limiting in our opinion, especially for translating the method for larger and more complex tissue applications. Another example from a recent publication from our group utilized the contraction of a bioprinted cellular collagen‐methacrylate structure to fuse it to a separately fabricated poly(L‐lactic) acid‐poly(lactide‐*co*‐glycolide) scaffold.^[^ ^51^ ^]^ Moreover, in certain circumstances, the contraction might be utilized to fabricate smaller constructs with higher resolution. However, the degree of contraction can vary substantially with cell type, cell density, cell passage, and hydrogel properties among other variables.^[^ ^52^, ^53^ ^]^ Therefore, we propose that further construct deformation might arise when printing multilayered tissues with different cell types since the layers will contract unevenly. Thus, we believe that our simple method of counteracting contraction of bioprinted constructs where the support is external and removable is desirable.

While cultivation in CarGrow does not completely inhibit the contraction in the described setup, certain modifications of the medium salt composition (Figure S5A–C, Supporting Information) or changes to the construct geometry and confinement in CarGrow (Figure S5D, Supporting Information) can be employed to further limit the contraction. Increasing the concentration of KCl results in a higher stiffness of the granular gel matrix, which has been shown to affect the contraction of a cellular beam.^[^ ^53^, ^54^ ^]^ Thus, the overall reduction in contraction is a function of the mechanical properties of CarGrow, the bioink, and the geometry of the construct.

To verify whether the post‐printing culture in CarGrow reduces the construct contraction for larger 3D geometries, we performed an additional experiment where HNDFs were printed in a diverging blood vessel structure using fibrinogen‐based bioink. The printed vessel‐like structures were either cultured within CarGrow or extracted to a liquid medium after printing. The extracted structures were washed thoroughly with medium to ensure a complete removal of the microgels, especially from the lumen. We examined and photographed the scaffold contraction during culture for 8 days. Figure 3D and Figure S6 in the Supporting Information show the pronounced difference in the dimensions of the constructs between treatments after 8 days of culture, further supporting our findings that CarGrow enhances the dimensional stability. To the best of our knowledge, this is the first research that fabricated and utilized kappa carrageenan microgels as granular bed to mechanically support cellular construct for days.

### 2.4 Print‐and‐Grow Approach Offers High Cellular Viability during Cultivation in CarGrow

**Figure 4:**
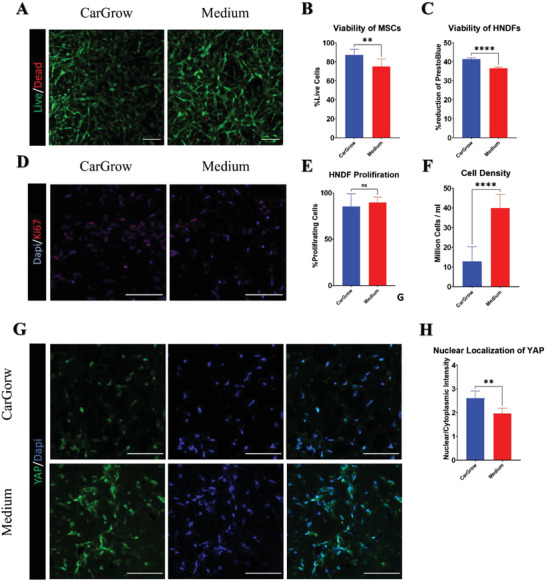
Cell viability and proliferation during culture in CarGrow. A) Live/dead assay of printed MSCs constructs 7 days after printing depicting live cells (green) and dead cells (red) (scale bar: 100 µm). B) Quantification of MSCs viability (*n* = 7). C) Percent reduction of PrestoBlue by printed HNDF constructs 7 days after printing (*n* = 5). D) Cryosections of printed HNDFs constructs 7 days after printing stained for ki67 with DAPI nuclear staining (scale bar: 100 µm). E) Quantification of ki67 marker as an indication for proliferation (*n* = 8). F) Cell density in printed HNDF constructs 7 days after printing as quantified from DAPI nuclear staining (*n* = 7). G) Cryosections of printed HNDFs constructs 7 days after printing stained for YAP with DAPI nuclear staining (scale bar: 100 µm). H) Quantification of the nuclear/cytoplasmic intensity of YAP stain (*n* = 6). ns: not significant, **p < 0.01, ****p < 0.0001.

As discussed above, to create functional tissues, the bioprinted constructs require a period of post‐printing cultivation. We have shown that it is possible to substantially preserve the shape of the tissue during this cultivation period by using the print‐and‐grow method; however, it is also essential that these constructs maintain their viability. Therefore, we sought to examine the viability of MSCs and HNDFs printed and grown within CarGrow. We printed constructs containing MSCs or HNDFs using a fibrin‐based bioink within CarGrow in a concentric disk shape with an outer and inner diameter of 7 and 3 mm, respectively, and a height of 2 mm. As before, the printed constructs were allowed to cross‐link for 30 min at 37 °C and were subsequently left to incubate in the same CarGrow or extracted immediately and transferred to a liquid medium to serve as a control. After 7 days of cultivation, we performed a live/dead staining assay on the constructs containing MSCs and calculated the percentage of live cells (**Figure** **4**A). We found that MSCs incubated in CarGrow showed significantly higher viability, with 87.1 ± 6% live cells compared to 75.2 ± 7.8% in the control group (Figure 4B). For HNDF‐containing constructs, we performed a colorimetric PrestoBlue viability assay after 7 days of cultivation. The results show that HNDFs incubated in CarGrow had a significantly higher reduction of PrestoBlue dye, indicating a higher cell viability compared to the control (Figure 4C). Lastly, to verify that these viable cells are proliferating and are not in a quiescent state, we stained HNDF‐containing constructs for proliferation marker ki67 and nuclei after 7 days of cultivation (Figure 4D). The images were analyzed and quantified for the percentage of ki67 positive cells. The results yielded a similar percentage of proliferating cells (≈86%) in constructs incubated in CarGrow or extracted from it (Figure 4E). These results indicate that cells incubated within CarGrow can retain their viability and proliferation capability while preserving their shape and structural stability. Furthermore, the percentage of live cells appeared to be higher in constructs cultivated in CarGrow as opposed to extracted constructs, indicating an additional advantage of using support materials in a continuous process of print‐and‐grow.

We hypothesized that the observed increase in viability could be related to the differences in the cell density, construct microstructure, and mechanical cues. The contraction of constructs leads to a significant reduction in the hydrogel volume and thus a higher cell density. Quantification of cell concentration in printed constructs incubated for 5 days reveals an average concentration of 12.9 ± 7.3 million cells per mL for constructs incubated in CarGrow, as opposed to 39.9 ± 6.9 million cells per mL for constructs extracted from CarGrow (Figure 4F). This approximately threefold increase of cell density might contribute to the difference in observed viability since there is a known correlation between cell density and cell viability in engineered tissues.^[^ ^30^ ^]^

In addition, the contraction leads to the compaction of the hydrogel network fibers and may thereby alter the porosity of the construct. We aimed to examine the porosity of constructs incubated in CarGrow or extracted to medium (Figure S7, Supporting Information). Constructs incubated in CarGrow had a smaller average pore area of 10.24 ± 3.5 µm^2^ compared to constructs extracted to medium with pore area of 17.79 ± 4.1 µm^2^ (Figure S7C, Supporting Information). Moreover, the density of pores in constructs cultivated in CarGrow (26 724 ± 3909 pores per mm^2^) was higher compared to the pore density in constructs extracted to medium (17 091 ± 3407 pores per mm^2^) (Figure S7D, Supporting Information). Although the pores are expected to get smaller in the contracted scaffolds, the average size of pore increased. We believe this might be due to the contraction of pores to a size smaller than the detection limit of the microscope. In addition, the pore walls are thicker in the medium‐incubated constructs as opposed to the CarGrow‐incubated constructs (Figure S7A, Supporting Information). Furthermore, the distribution of pore sizes within the constructs incubated in CarGrow is narrower than that of the constructs incubated in medium (Figure S7E, Supporting Information). These results indicate different pore sizes and distribution in the Car Grow‐incubated construct, which may provide for better nutrient/waste exchange within the construct, thus, increasing the overall cell viability.^[^ ^55^, ^56^, ^57^ ^]^

Furthermore, due to the different contraction behavior of constructs incubated in CarGrow, we hypothesized that the cells receive different mechanical signals as well. To examine this assumption, we stained cryo‐sections of printed HNDFs in fibrin gels for Yes associated protein (YAP) expression (Figure 4G). YAP is a transcription factor involved in the mechanotransduction of mechanical forces in many cell types.^[^ ^58^, ^59^ ^]^ Its nuclear localization is associated with activation of the mechanotransduction pathway, which can lead to enhanced viability of the constructs.^[^ ^60^, ^61^ ^]^ We observed a higher nuclear localization of YAP in constructs incubated in CarGrow compared to those that were extracted (Figure 4H). This result indicates that the cells cultivated within CarGrow receive different mechanical cues compare to extracted scaffolds. During the contraction of the ring‐shaped constructs, the contractile forces of the cells are exerted on the hydrogel and lead to a total reduction of the inner and outer diameters. In CarGrow‐incubated constructs, the contraction of the inner diameter compresses the microgels in the construct's lumen, exerting an inward radial stress on it. If the stresses that the cell apply do not exceed the yield strength of the microgels, the microgels will exert an equal and opposite outward radial stress on the cellular hydrogel. Due to the ring‐shape of the construct, the application of a distributed force on the inner lumen leads to the formation of circumferential (hoop) and radial stresses in the hydrogel, that can be described using Lamé’s equations for thick‐walled pressure vessels^[^ ^62^ ^]^

(4) $$\mathrm{Circumferential}\;\mathrm{stress}:\;\sigma_{h}=\frac{p_{i}r_{i}^{2}-p_{o}r_{o}^{2}}{r_{o}^{2}-r_{i}^{2}}+\frac{\left(p_{i}-p_{o}\right)r_{o}^{2}r_{i}^{2}}{\left(r_{o}^{2}-r_{i}^{2}\right)r^{2}}$$

(5) $$\mathrm{Radial}\;\mathrm{stress}:\;\sigma_{r}=\frac{p_{i}r_{i}^{2}-p_{o}r_{o}^{2}}{r_{o}^{2}-r_{i}^{2}}-\frac{\left(p_{i}-p_{o}\right)r_{o}^{2}r_{i}^{2}}{\left(r_{o}^{2}-r_{i}^{2}\right)r^{2}}$$

where *σ*_h_, *σ*_r_ are the circumferential and radial stresses, respectively, *r*_o_, *r*_i_ are the outer and inner radii, respectively, *r* is the variable radius, and *p*_i_,*p*_o_ are the internal and external pressures in the ring, respectively. The internal pressure in CarGrow‐incubated constructs is due to the radial stresses that the microgels exert on the hydrogel. According to these equations, in constructs incubated in medium, there is no difference in pressure between the inner and outer sides, and thus there are no additional circumferential and radial stresses. In CarGrow‐incubated constructs, the pressure difference leads to the formation of stress fields that vary with the radial location. Cells suspended in the hydrogel can sense these stresses which activate the YAP pathway. Enhanced mechanical signaling and YAP nuclear localization has been correlated with enhanced viability.^[^ ^63^ ^]^

The results presented above demonstrate the advantages of using the print‐and‐grow method to promote shape stability and viability of the bioprinted constructs. This result supports our hypothesis and demonstrates that cells can be cultivated within support material for a prolonged period without hampering their viability and proliferation ability.

### 2.5 Print‐and‐Grow Approach Enables the Printing of Functional Tissues

**Figure 5:**
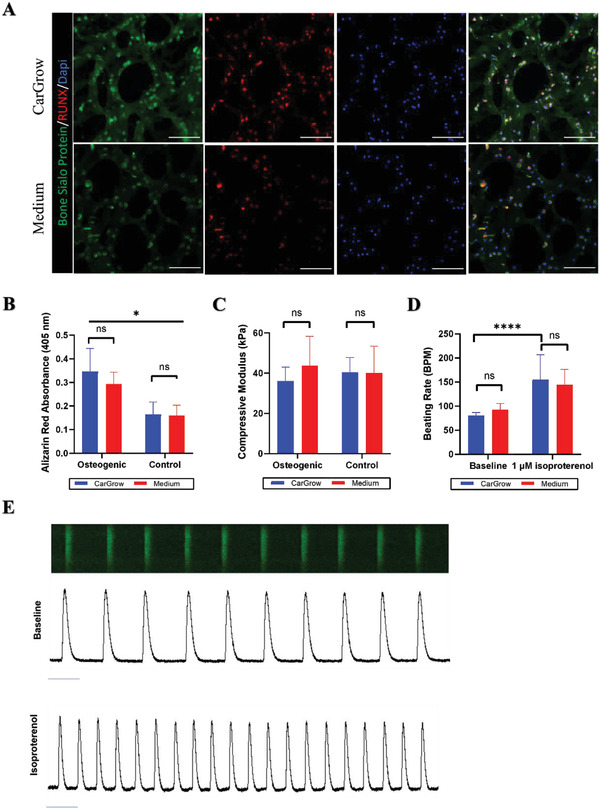
Cell differentiation and function during culture in CarGrow. A) Cryosections of printed MSCs constructs 7 days after printing stained for BSP‐2 (green), RUNX2 (red) with DAPI nuclear staining (blue). (Scale bar: 100 µm.) B) MSC osteogenesis induction in CarGrow as measured by quantification of mineral content (*n* = 3). C) Compressive modulus of printed MSC construct 7 days after printing (*n* = 3). D) Average cardiomyocytes beating rate as measured from the calcium‐flux imaging (*n* = 11). E) Representative fluorescent signal of beating cardiomyocytes. (Scale bar: 1 s). ns: not significant, *p < 0.05, ****p < 0.0001.

Using tissue‐specific stem and precursor cells is common in various tissue engineering applications. These cells must differentiate and mature to the desired cell type to become a functional tissue. We have shown that applying the method of print‐and‐grow leads to significantly enhanced shape stability of printed constructs while offering a favorable growth environment for cell viability. Next, we aimed to examine if incubation of the bioprinted construct within CarGrow can affect the differentiation capability of stem cells or affect the function of committed cells. To verify the differentiation capability of cells cultivated in CarGrow, we printed undifferentiated MSC‐laden fibrin bioink constructs and incubated them inside CarGrow. After cross‐linking, osteogenic medium was gently added on top of the support material. Control constructs were extracted into a liquid osteogenic medium. The osteogenic medium induces the differentiation of MSCs into osteoblasts. After 7 days of cultivation, some of the constructs were fixed and cryo‐sectioned for immunofluorescent staining. We stained the constructs for nuclear osteogenic marker RUNX and cytoplasmic osteogenic marker BSP‐2.^[^ ^64^ ^]^ The resulting images show similar staining of the osteogenic markers in both groups (**Figure** **5**A). This indicates that the cells cultivated in the support material could start differentiating into an osteoblastic lineage. Osteoblasts build the bone‐specific ECM, which contains calcium phosphate minerals.^[^ ^65^ ^]^ Thus, we aimed to measure the mineral content of constructs cultivated in CarGrow as a measure of the differentiation potential of the encapsulated cells. Constructs were stained with alizarin red dye, which was later extracted and quantified to measure calcium content. Constructs supplied with a control nonosteogenic medium served as a control. The results show that the mineral content of osteogenic constructs cultivated in CarGrow and those extracted from CarGrow is similar (Figure 5B). Furthermore, the mineral content of osteogenic constructs of both groups was significantly higher than the mineral content of control constructs without osteogenic differentiation. Lastly, the mechanical properties of osteogenic printed constructs were evaluated to examine the effect of print‐and‐grow approach on the compressive stiffness. After 7 days of cultivation either within the support material or liquid medium, constructs were imaged to measure their approximate cross‐sectional area, and immediately subjected to a uniaxial compression test before fixation. The stresses and strains were calculated for each construct, and the slope of the curves in the linear range was calculated; this slope is indicative to the compressive modulus of the constructs (Figure 5C). No significant difference between the compressive modulus of the different groups was observed. Taken together, the above results show that using the print‐and grow method does not negatively impact the differentiation potential and functionality of encapsulated MSCs. Future work should explore the differentiation capability of other stem cell types using print‐and‐grow.

Recently, significant progress has been made in the field of 3D bioprinting of heart tissue.^[^ ^10^, ^66^ ^]^ We sought to examine the suitability of the print‐and‐grow approach for creating functional cardiac constructs. Induced pluripotent stem cell (iPSC)‐derived cardiomyocytes were bioprinted and cultivated within CarGrow. Their functionality was evaluated using genetically encoded calcium indicator: GFP‐coupled calmodulin (GCaMP), which indicates intracellular calcium flux related to cardiomyocytes contractions. The cells were suspended in a fibrin‐based bioink and printed in a wave pattern (Figure S8, Supporting Information). After cross‐linking, medium was added gently to constructs cultivated in CarGrow, while constructs extracted to liquid medium were used as a control. After 5 days of incubation, we live imaged the constructs and observed the characteristic rhythmic calcium flux and contraction (Figure S8B, Supporting Information). The acquired data were subsequently analyzed, and the beating rate was calculated (Figure 5D,E). Cardiomyocytes in both groups showed a similar beating rate, indicating that print‐and‐grow does not interfere with the cardiomyocytes functionality, moreover it enables their live imaging within CarGrow. Next, we performed an additional functionality test by exposing the cardiomyocytes to isoproterenol, an adrenaline analog that can accelerate the action potential of the cells and subsequently accelerate the beating rate. We added isoproterenol to the medium of printed constructs and recorded the fluorescent signal associated with the calcium flux (Movie S2, Supporting Information and Figure 5D,E). The results indicate that cells in both groups responded to the isoproterenol by increasing their beating rate. Thus, the functionality of cardiomyocytes is preserved when using print‐and‐grow.

Overall, these results demonstrate the potential of using print‐and‐grow to engineer functional and viable tissues that can be directly imaged while maintaining shape fidelity to the patient‐specific design.

## 3 Conclusion

The present work introduces a novel concept of 3D bioprinting and subsequent cultivation of cellular constructs in a microgel support material: print‐and‐grow. Using a facile and reproducible method, we fabricated *κ*‐Carrageenan‐based microgels (CarGrow), which serve as an ultimate support material for the print‐and‐grow process. The microstructure and rheological properties of CarGrow microgels enable high fidelity 3D bioprinting using a broad range of cell‐friendly low viscosity bioinks. The transparency of CarGrow allows direct monitoring of the bioprinting process and live imaging of cellular constructs during the post‐printing cultivation phase. Cellular constructs cultivated in CarGrow substantially preserved their size and shape over time. Moreover, CarGrow promoted cell viability and provided suitable conditions for differentiation of the cellular scaffolds.

Overall, print‐and‐grow allows fabrication of functional tissue with a desired shape and size in a reliable and reproducible manner. Our approach may lead to emergence of universal and user‐friendly 3D bioprinting technology for creating tissue implants and in vitro tissue modeling. Furthermore, CarGrow has a potential to benefit the 3D printing for cultured meat production, because its main component *κ*‐Carrageenan is a Food and Drug Administration‐approved food additive. We envision that further development of the print‐and‐grow approach may transform 3D bioprinting to applicable technology for regenerative medicine, drug discovery, and food industry.

## 4 Experimental Section

### CarGrow Microgels Preparation

*κ*‐ Carrageenan microgels were prepared using W/O emulsion technique. 1.5% w/v *κ*‐Carrageenan (Sigma Aldrich) was slowly added into a 90 mL hot solution (83 °C) of   0.1m potassium chloride (Merck) for 1 h under magnetic stirring. Separately, 0.2% v/v of Span 80 (Sigma Aldrich) was added to 450 mL of paraffin oil (Sigma Aldrich) and heated to 83 °C. After 1 h, the *κ*‐Carrageenan solution was added to the oil phase and stirred using an overhead stirrer at 1500 RPM for 10 min. Then, the emulsion was allowed to cool to room temperature under continuous overhead stirring at 600 RPM. After cooling, the microgel suspension was transferred into 50 mL tubes and centrifuge at 3000 RCF for 5 min to separate the microgel and oil phases. The supernatant of the oil phase was removed and replaced with 70% ethanol. The samples were vortexed vigorously to resuspend the microgels and were centrifuged at 3000 RCF for 5 min. This step was repeated three times until most of the paraffin oil was removed. Next, the sedimented microgels were suspended in   0.1m KCl solution and centrifuged at 3000 RCF for 5 min. This wash step was repeated two times to remove the remaining ethanol. Next, to remove aggregates, the samples were suspended with   0.1m KCl and filtered using Buchner funnel (60 µm pore nylon filter, Merck). At this stage, the filtered samples were stored at 4 °C for later use. For visualization of the microgels after the fabrication process, photographs of dilute samples were taken using an inverted phase‐contrast microscope (Nikon eclipse TS100). Prior to printing, the microgels were suspended in the relevant cross‐linking solution suitable to the selected bioink: 0.1% w/v calcium chloride (CaCl_2_, J.T.Baker), 12 U mL^−1^ thrombin (Evicel) in 1XPBS or 1XPBS for alginate, fibrin, and GelMA, respectively. Next, the samples were vortexed vigorously and centrifuged at 3000 RCF for 5 min. The supernatant was removed, and the microgels were centrifuged again at 3000 RCF for 2 min to remove the remaining fluid. Lastly, the CarGrow microgels were transferred into 35 mm culture dishes or 24‐well plates using a positive displacement pipette (Microman, Gilson). For cell printing, the filtered samples were sterilized three times with 70% ethanol and centrifuged at 3000 RCF for 5 min. Next, the samples were washed twice with sterile 0.1m KCl and centrifuged at 3000 RCF for 5 min. Then, two washes were performed using sterile 1XPBS and Dulbecco's modified Eagle medium (DMEM) low glucose, respectively, and the samples were centrifuged at 3000 RCF for 5 min. The last wash before cell printing was performed using the cross‐linking solution for the fibrin bioink: 12 U mL^−1^ thrombin in DMEM low glucose and centrifuged at 3000 RCF for 5 min. The supernatant was removed, and the sample was centrifuged again at 3000 RCF for 2 min to remove the remaining fluid.

### Size Distribution Measurements

The size distribution of CarGrow was measured using a Malvern Mastersizer 2000. The microgel samples were diluted in   0.1m KCl and vortexed vigorously to obtain a homogenous dispersion before the measurements. Six independent fabrications from different batches were measured, and the average size was calculated.

### Structure Characterization using Cryo‐SEM

Cryo‐SEM experiments were performed using a Zeiss Ultra Plus high‐resolution SEM (HR‐SEM), equipped with a Schottky field‐emission gun and with a BalTec VCT100 cold‐stage maintained below −145 °C. Specimens were imaged at low acceleration voltages of 1 kV and working distances of 3–5 mm. Everhart Thornley (“SE2”) secondary electron imaging detectors were used. Low‐dose imaging was applied to the specimen to minimize radiation damages. Specimens were prepared by the drop plunging method, a 3 µL drop of solution was set on top of a special planchette maintaining its droplet shape and was manually plunged into liquid ethane, after which it was set on top of a specialized sample table. The frozen droplets were transferred into the BAF060 freeze‐fracture system, where they were fractured by a rapid stroke from a cooled knife, exposing the inner part of the drop. They were then transferred into the pre‐cooled HR‐SEM as described above. Ideally, imaging was performed as close as possible to the drop surface, where the cooling rate should be maximal.

### Rheological Measurements

Rheological measurements were performed using MCR 302 rheometer (Anton paar) with parallel plate geometry (25 mm diameter) and 1 mm gap. All samples (*n* = 3) were centrifuged at 3000 RCF for 5 min, and the supernatant was removed. Then, the samples were centrifuged again at 3000 RCF for 2 min to remove the remaining fluid. Next, 600 µL of the microgels was loaded on a rheometer plate for the measurements.

To examine the LVR, amplitude sweep experiments were performed. The storage (*G'*) and loss (*G''*) modulus were measured in the range of 0.01%–100% strain, under oscillation frequency of 10 Hz. Temperature sweep experiments were performed to examine the temperature sensitivity of the microgels. The *G*' and *G''* were measured at a wide range of temperatures (20–50 °C) under *ω* = 10 s^−1^ and 0.1% amplitude strain which was within the LVR. Steady shear flow measurements were used to study the shear‐thinning behavior of CarGrow. The viscosity was measured in a wide range of shear rates, 0.001–100 s^−1^, using logarithmic ramp. Recovery measurements were performed to investigate the ability of the material to self‐heal after structure breakdown using oscillatory experiment. The *G'* and *G''* were measured at a low strain percentage of 0.1% followed by high strain percentage of 100% for three cycles ending by additional low strain step.

### FRAP Analysis

FRAP was performed using LSM700 confocal microscope (Zeiss). 0.1% w/v of fluorescein sodium salt (376 MW, Sigma Aldrich) or Albumin‐FITC (66.4 kDa, Sigma Aldrich) were dissolved in 1XPBS. Samples of CarGrow, gelatin support bath (LifeSupport, Fluid Form), or PBS were prepared and mixed with fluorescein or Albumin‐FITC tracer solution in 1:1 ratio. The samples were vortex, and 100 µL from each sample was transferred into 96‐well plates. The FRAP experiment was performed by bleaching a circular area for 50 iterations. The diffusion coefficients were calculated using the open‐source MATLAB code “frap_analysis.”^[^ ^67^ ^]^

### Transparency Test

To demonstrate and quantify the transparency of CarGrow, absorbance measurements were performed using UV‐Visible spectrophotometer Synergy HTBioTek (BioTek Instruments, Winooski, VT, USA) at wavelengths of 400–700 nm. The results were compared to a commercially available turbid support material composed of gelatin particles (Life support, Fluid Form). The samples were prepared and centrifuged as mentioned above, or according to the manufacturer protocol, and transferred into 24‐well plate for the measurements. The transmittance spectra were calculated (Figure S1, Supporting Information) using the logarithmic relation

(6) $$\mathrm{Transmittance}\;\left[\%\right]=10^{\left(2-\mathrm{Absorbance}\right)}$$

### Bioinks Preparation

2% w/v alginate (medium viscosity, Sigma‐Aldrich) was dispersed in deionized water with 0.1% w/v Alcian blue (Alfa Aesar) for visualization. The solution was stirred overnight using magnetic stirring. GelMA bioink was prepared first by dispersing 10% w/v of lyophilized GelMA stock (Allevi) in PBS at 37 °C overnight. 0.2% lithium phenyl‐2,4,6‐trimethylbenzoylphosphinate (LAP, Sigma‐Aldrich) and 1.6% polyethylene oxide (PEO, ACROS Organics) were dispersed separately in 1XPBS at 37 °C overnight. To obtain a 5% w/v GelMA bioink, the GelMA and the LAP‐PEO solutions were mixed in a 1:1 ratio and stored at 4 °C for 15 min prior to printing. Collagen bioink was prepared by neutralizing a solution of Collagen I from rat tail (Corning) according to the manufacturer instructions. Next, the collagen solution was mixed with 1.6% w/v PEO (ACROS Organics) solution in a 1:1 ratio to obtain a final collagen‐PEO bioink with 5 mg mL^−1^ collagen I and 0.8% w/v PEO. Fibrin bioink was prepared as described previously with slight changes and included several steps.^[^ ^33^ ^]^ For experiments including cells, 3 mg mL^−1^ hyaluronic acid (Sigma‐Aldrich) was first dissolved in DMEM low glucose medium supplemented with 1% pen/strep and 30 KIU mL^−1^ aprotinin. For experiments without cells, 3 mg mL^−1^ hyaluronic acid was dissolved in DMEM low glucose medium supplemented with 10% glycerol (Sigma‐Aldrich). Next, 30 mg mL^−1^ type A gelatin (Sigma‐Aldrich) was added and mixed overnight at 37 °C. Finally, 10 mg mL^−1^ fibrinogen (Seqens in vitro diagnostic) was added, and the solution was mixed until complete dissolution. The solution was then sterile filtered and stored at −20 °C until use. Prior to use, the bioink was thawed at 37 °C, and cells were suspended in the bioink at a concentration of 6 million cells per 1 mL of bioink. Prior to cell mixing, bioinks were centrifuged for 20 s at 3000 *g* before use to remove air bubbles. Where necessary, 0.5 µm fluorescent microspheres (Polysciences) were mixed at 1:100 ratio with the bioink to aid fluorescent imaging.

### 3D Printing of Rectilinear Patterns and Large Constructs

Prepared bioinks were transferred into 3 mL printing cartridges (Nordson EFD) fitted with 300 µm inner diameter blunt end needle (CML supply). The printing cartridge was then loaded into a printing tool of 6 axis extrusion bioprinter BioAssemblyBot (Advanced Solutions). The design of the printed constructs was performed using TSIM (Advanced Solutions) or SolidWorks (Dassault Systèmes SolidWorks Corp., USA). The object was sliced to the desired slice thickness (80% or 50% of the needle's inner diameter), and the file was sent to the bioprinter. The printing was performed with specified pressure and speed parameters as determined by volumetric calibration of the printing parameters for each bioink. The volumetric calibration was performed by extruding bioink at a specified pressure for a set amount of time and measuring the extruded volume. Based on the principle of volume conservation and the following equation, the optimal printing speed was calculated

(7) $$\mathrm{Volume}\left(\mathrm{droplet}\right)=\mathrm{Volume}\left(\mathrm{filament}\right)=\pi {\left(\frac{d}{2}\right)}^{2}\cdot \mathrm{speed}\cdot \mathrm{time}$$

The range of printing parameters for each type of bioink is summarized in **Table** **1**. After printing, the constructs were allowed to fully cross‐link for 30 min, followed by either medium addition or construct extraction by gentle PBS pipetting and dilution of the support material.

**Table 1:** Printing parameters for the bioinks used

Bioink	Pressure [psi]	Speed [mm s^−1^]
Alginate	5–10	10–15
Fibrin	6–12	10–15
Gel‐MA	1.5–3	20–25
Collagen	1.5–3	17.5–20

### Printability Index Calculation

The printability index for square shape was calculated using the following equation^[^ ^47^ ^]^

(8) $$P_{r}=\frac{L^{2}}{16A}$$

where *L* is the perimeter and *A* is the area of the square. For each bioink with a rectilinear pattern, the perimeter and the area of the internal square were measured using FIJI ImageJ, and the Pr index was calculated (*n* = 3). Statistical comparison was performed between the different bioinks.

### Cell Culture

Cells are thawed and seeded on 75 or 150 cm^2^ cell culture flasks at a density of 10 000 cells per cm^2^ (Techno Plastic Products AG, Switzerland). All cell culture was performed in humidified incubators at 37 °C, and an HEPA‐filtered atmosphere of air and 5% of CO_2_. Medium changes were performed every 2–3 days, except where indicated otherwise. Human neonatal dermal fibroblasts (HNDFs, Lonza Wakersville Inc., USA), or red fluorescent protein‐expressing HNDF (HNDF‐RFP, Angio‐Proteomie, USA) were cultured in endothelial cell medium (ScienceCell) supplemented with 5% fetal bovine serum (FBS, ScienceCell) and endothelial cell growth bullet kit (ScienceCell). MSCs (Lonza) were cultured in Nutristem medium (Biological Industries). Cells were harvested for experiments during passages 4–7 at ≈70% of confluence. For live cell tracking of MSCs or HNDF, cells were labeled with either DiI, DiD, or DiO Vybrant cell labeling solution (Thermo Fisher). For osteogenic differentiation of MSCs, DMEM low‐glucose medium was supplemented with 10% FBS, 1% Pen‐Strep, 100 × 10^−9^ m dexamethasone, 10 × 10^−3^ m *β*‐glycerol phosphate, and 50 × 10^−6^ m ascorbic acid.

### hiPSCs Culture and Directed Cardiac Monolayer Differentiation

In this study, human induced pluripotent stem cells (hiPSC) expressing GCaMP (kindly gifted by Prof. Bruce Conklin, Gladstone Institute, USA) were cultured on Matrigel coated plates with mTeSR‐1 medium (StemCell Technologies). hiPSC were passaged by using 0.5 × 10^−3^ m ethylenediaminetetraacetic acid (Gibco) every 4–5 days. Differentiation of hiPSCs to cardiomyocytes was based on a previously described protocol.^[^ ^68^ ^]^ Briefly, at 80–90% confluence, the medium was exchanged with a differentiation medium composed of RPMI‐1640, 2% B27 supplement minus insulin (ThermoFisher Scientific), 1% penicillin/streptomycin, and supplemented with 6 × 10^−6^ m CHIR99021 (Stemgent) for 2 days. Medium was then exchanged into RPMI/B27 supplemented with 2 × 10^−6^ m Wnt‐C59 (Selleck Chemicals) for another 2 days. After the 5th day, cells were cultured with RPMI/B27 medium alone. Cardiomyocytes (after 10–14 days of differentiation) were enzymatically dissociated using TrypLE express (Gibco). For bioprinting experiments, 8 million cardiomyocytes were suspended in 1 mL fibrin bioink.

### Live‐Cell Imaging of Printed Cellular Scaffolds and Quantification of Contraction

Disk shape cellular constructs were printed and incubated in CarGrow or in a medium. HNDF constructs were labeled using DiO and MSC constructs were labeled with DiD. The constructs were imaged daily for 5 days after printing, using a brightfield fluorescent microscope (Axiovert 7, Zeiss). The inner and outer diameters of the constructs were measured using ZEN software, and the total area was calculated and normalized to the initial area after printing. Diverging blood vessel constructs were printed and incubated in CarGrow or a medium. Photographs were taken after printing, 1 day, 3 days, and 8 days.

### Viability Assays

Live/dead cell viability assay (Invitrogen) was performed according to the manufacturer's instructions. Briefly, 7 days following incubation, bioprinted constructs were washed once with PBS followed by incubation with calcein‐AM and ethidium homodimer‐1 for 45 min at 37 °C. The constructs were then imaged using Zeiss LSM700 confocal microscope. The images were analyzed using FIJI ImageJ, and the number of live and dead cells was counted, and the percentage of live cells was calculated. The PrestoBlue cell viability assay (Invitrogen) was performed on 7 days HNDF constructs by incubating them with the PrestoBlue reagent for 60 min. The absorbance was measured using a plate reader, and the percent reduction of PrestoBlue was calculated according to the manufacturer instructions

### Immunofluorescence and Histological Staining

Whole scaffolds were fixated in 4% paraformaldehyde (PFA; Electron Microscopy Sciences, USA) for 20 min, and then washed three times with PBS, 5 min each wash. For tissue sectioning, the samples were immersed in 30% w/v sucrose solution overnight at 4 °C following fixation. Then, the constructs were embedded in optimal cutting temperature compound (TissueTek) and frozen at −20 °C for cryo sectioning (10 µm thickness). The sections were permeabilized using 0.5% Tween 20, washed three times with PBS, blocked with 5% bovine serum albumin solution, and incubated at 4 °C overnight with either rabbit anti‐ki67 (1:100, abcam ab15580), mouse anti‐YAP (1:100, santa cruz sc‐376830), rabbit anti‐BSP2 (1:200, MilliporeSigma AB1854), or anti‐human RUNX2 (1:100, santa cruz sc‐390351). The sections were washed thrice in PBS and incubated for 3 h at room temperature with a secondary antibody in PBS solution consisting of donkey anti‐mouse Alexa‐647 (1:400, Jackson 715‐605‐151) and donkey anti‐rabbit Cy3 (1:400, Jackson, 715‐605‐152) mixed with 4′,6‐diamidino‐2‐phenylindole (DAPI, 1:1000, Sigma).

### Slides and Constructs Imaging

The constructs were imaged using LSM700 confocal microscope or Axiovert 7 (Zeiss, Germany). Image analysis was done using Zen software (Zeiss, Germany) or FIJI ImageJ. Macroscopic photographs were acquired using a Samsung S20 smartphone.

### Pore Size Quantification

10 µm thick cryosections of printed HNDF constructs were stained with hematoxylin and eosin to aid pore visualization. The images were segmented by thresholding so that the regions within that tissue that are not stained (i.e., white areas) are marked as pores, and their area calculated.

### Mechanical Characterization of Printed Constructs

Compression tests of printed MSC constructs were done using an AR‐G2 rheometer (TA Instruments, USA) equipped with a 20 mm parallel plate geometry. Nonfixed samples of printed MSC constructs were imaged to calculate their cross‐sectional area and loaded in the middle of the rheometer plate. Then, the constructs were compressed to 25% their original thickness. Stress–strain curves were calculated, and Young's modulus was obtained from the semilinear region of 20–40% strain.

### Alizarin Red Quantification Assay

Quantification of the mineral content of printed MSC constructs was performed using Alizarin Red S Staining Quantification Assay (Sciencell). Briefly, constructs were fixed using 4% PFA, followed by five washes with deionized water. Then, the constructs were incubated with the alizarin red solution, followed by dye extraction and measurement of absorbance according to the manufacturer's instructions.

### Optical Imaging GCaMP‐Expressing hiPSC‐CMs

To measure the beating rate of the GCaMP expressing cardiomyocytes within the printed tissue, live cell imaging was performed using the line‐scan mode of a Zeiss LSM700 laser‐scanning confocal microscope (Zeiss). To assess the beating response to adrenaline analog, the constructs medium was supplemented with 1 × 10^−6^ m isoproterenol (Sigma‐Aldrich). Recording of the calcium transients was performed 10 min after the addition of the isoproterenol. The GCaMP fluorescent recordings were analyzed using the Clampfit10.7 program (Molecular Devices) and the beating rate was subsequently evaluated.

### Statistical Analysis

Quantitative results were obtained from a minimum of three independent samples. Statistical analyses were performed using Prism 9. Two‐group comparisons were made using’ Student's *t*‐test, and multiple‐group comparisons were made using one‐way analysis of variance with Tukey's test for post hoc analysis. All data were presented as mean ± SD, and significance levels were presented as follows: *p < 0.05, **p < 0.01, ***p < 0.001, ****p < 0.0001.

## Conflict of Interest

The authors declare no conflict of interest.

## Supporting information

Supporting Information

Supplemental Movie 1

Supplemental Movie 2

## Data Availability

The data that support the findings of this study are available from the corresponding author upon reasonable request.
