# Supplementary material for: Print‐and‐Grow within a Novel Support Material for 3D Bioprinting and Post‐Printing Tissue Growth
Source: Adv Sci (Weinh). 2022 Oct 19;9(34):2200882. doi: 10.1002/advs.202200882 (PMC9731703; doi:10.1002/advs.202200882)
Supplement: Supplementary file 1 — Supporting Information [file ADVS-9-2200882-s001.pdf]

Supporting Information

Print-and-grow within a novel support material for 3D bioprinting and post-printing tissue growth

Majd Machour†, Noy Hen†, Idit Goldfracht, Dina Safina, Maya Davidovich-Pinhas, Havazelet Bianco-Peled, Shulamit Levenberg**

†The authors M.M. and N.H. made equal contribution to the work.

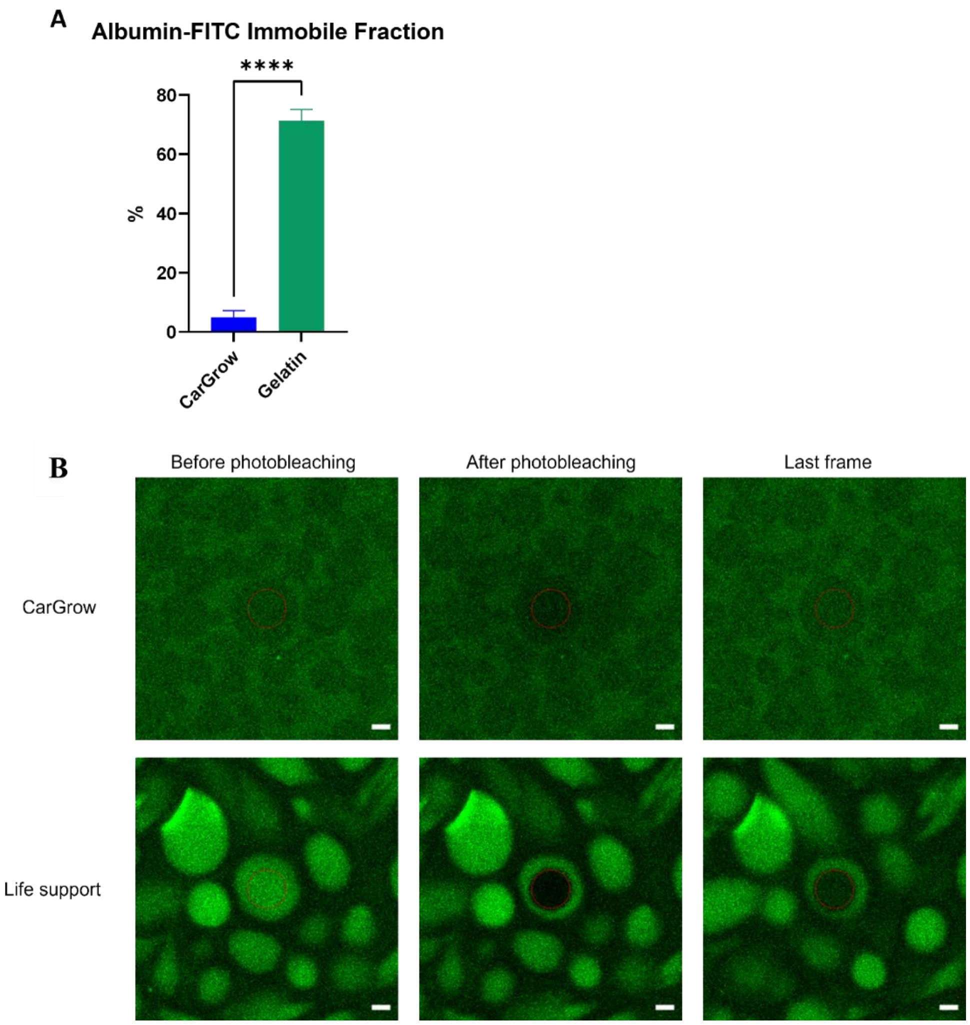

Figure S1. Single particle diffusion test. (A) Immobile fraction of Albumin-FITC tracers in CarGrow and LifeSupport as measured by fluorescent recovery after photobleaching (FRAP). (B) FRAP images of Albumin-FITC (66.4 KDa) tracer within CarGrow or LifeSupport granular materials. The images demonstrate full recovery of the bleach area (red circle) in CarGrow microgels (scale bar: 10µm).

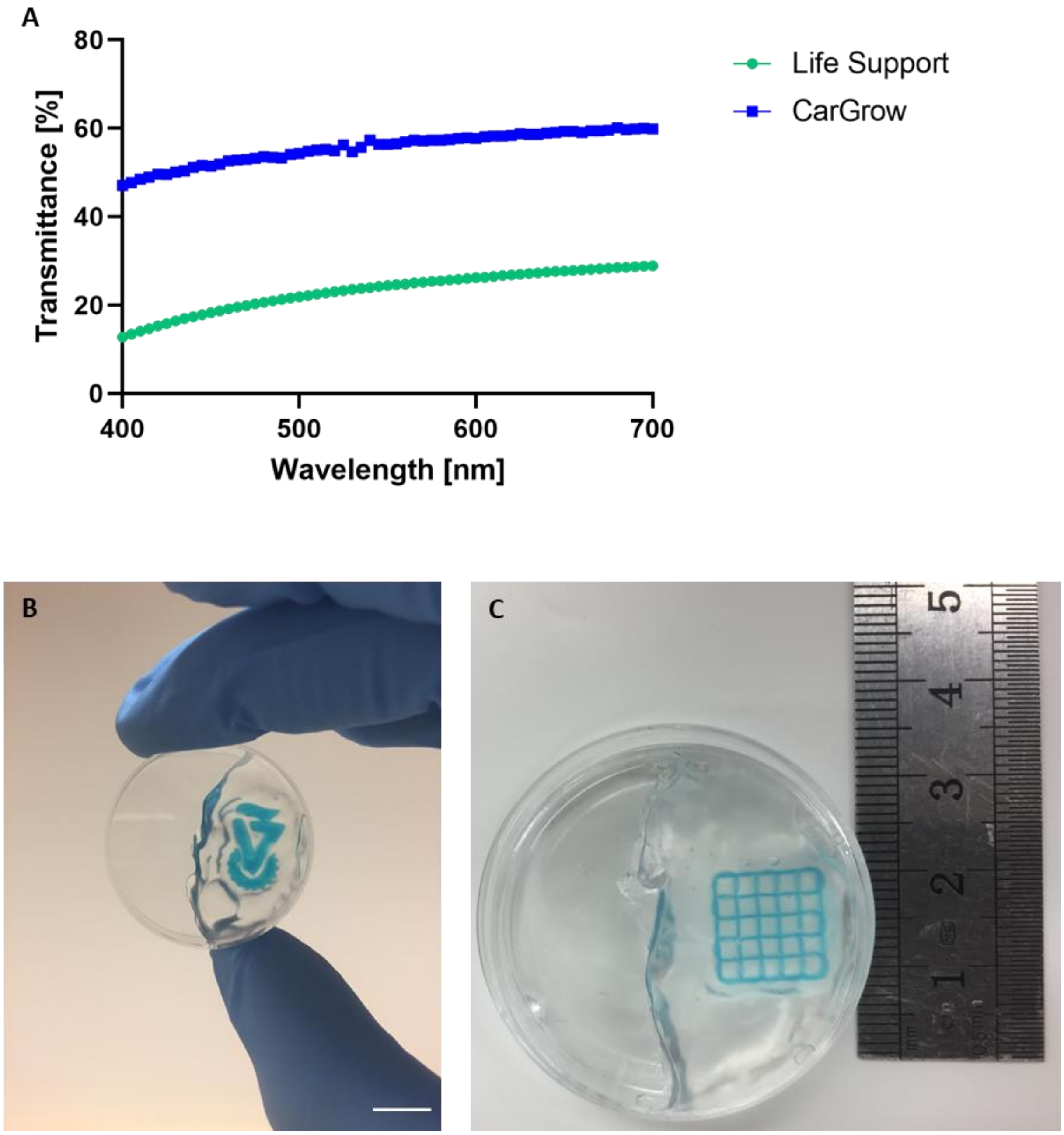

Figure S2. CarGrow transparency. (A) Transmittance curves of CarGrow microgels and gelatin support material (Life support). Printed structures of (B) Technion logo and (C) rectilinear pattern using 2% alginate with alcian blue dye (scale bar: 10 mm).

A

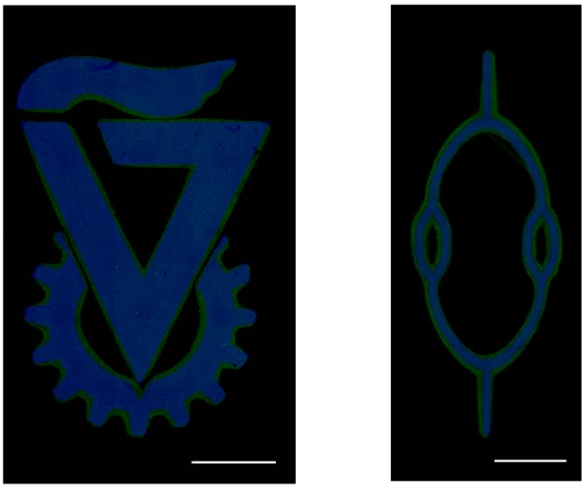

B

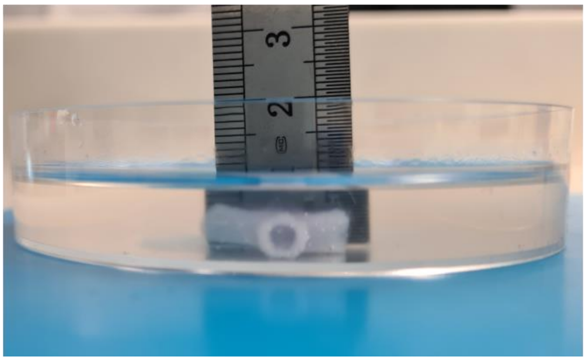

Figure S3. High resolution and shape fidelity of complex structures. (A) Overlay of CAD design (blue) and printed alginate constructs (green) showing the fidelity of the printing process (scale bar: 5 mm). (B) Photograph of the hollow vessel in PBS, after extraction from CarGrow. Fibrin based bioink was used to print hollow vessel structure.

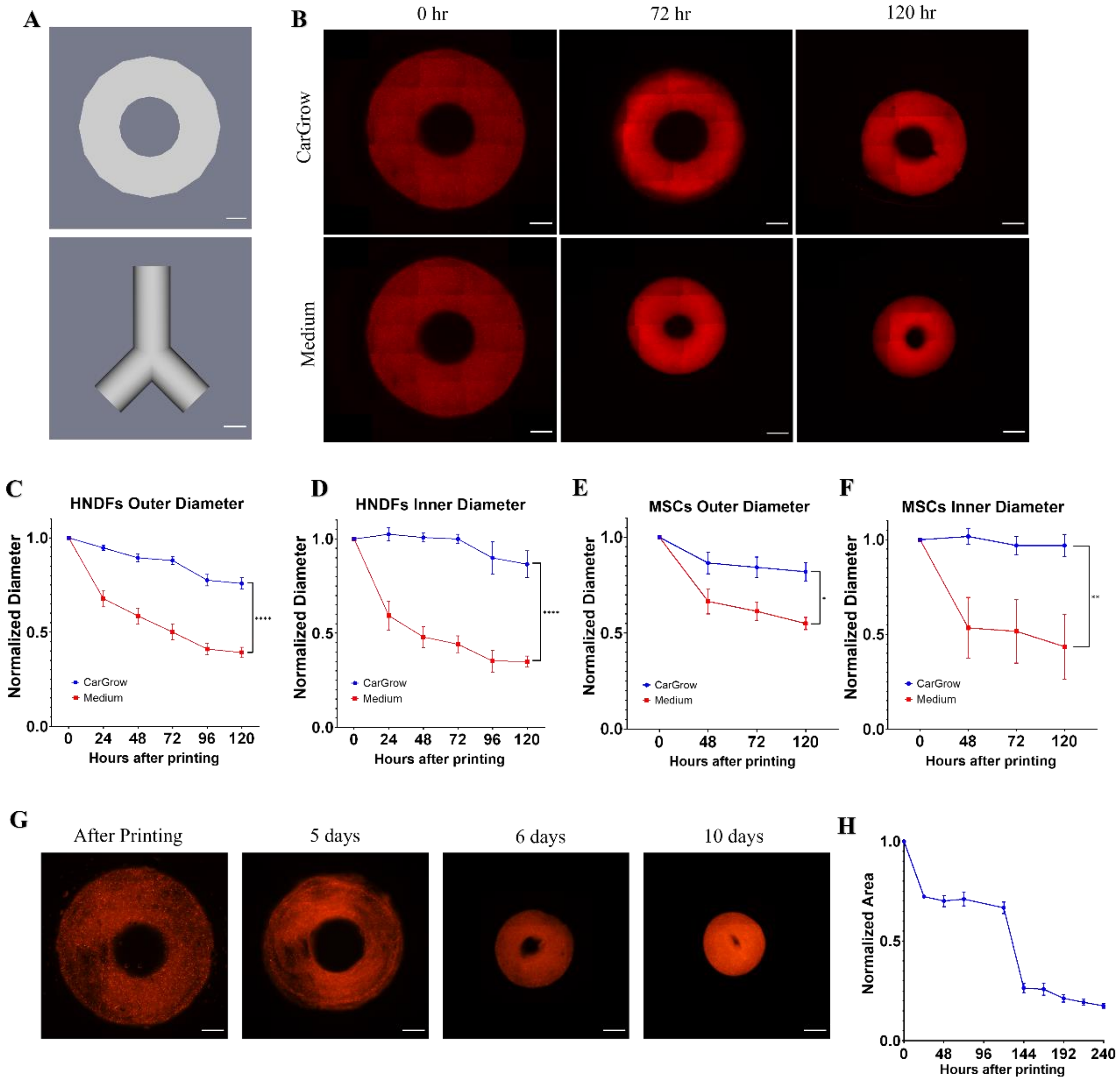

Figure S4. Incubation in CarGrow reduces cellular construct contraction. (A) Computer designs for printed cellular objects. (scale bar (top):1 mm, scale bar (bottom): 2 mm). (B) Fluorescence images of printed MSC constructs extracted to liquid medium or cultivated in CarGrow at different times after printing. (scale bar: 1 mm). (C) Quantification of the normalized outer diameter of printed HNDF constructs during incubation (n=6). (D) Quantification of the normalized inner diameter of printed HNDF constructs (n=6). (E) Quantification of the normalized outer diameter of printed MSC constructs during incubation. (n=8). (F) Quantification of the normalized inner diameter of printed MSC constructs (n=8). (G) Fluorescence images of printed HNDF-RFP constructs cultivated in CarGrow for five days and then extracted to liquid medium and cultivated for an additional five days (scale bar: 1 mm). (H) Quantification of the normalized area of printed constructs shown in (G).

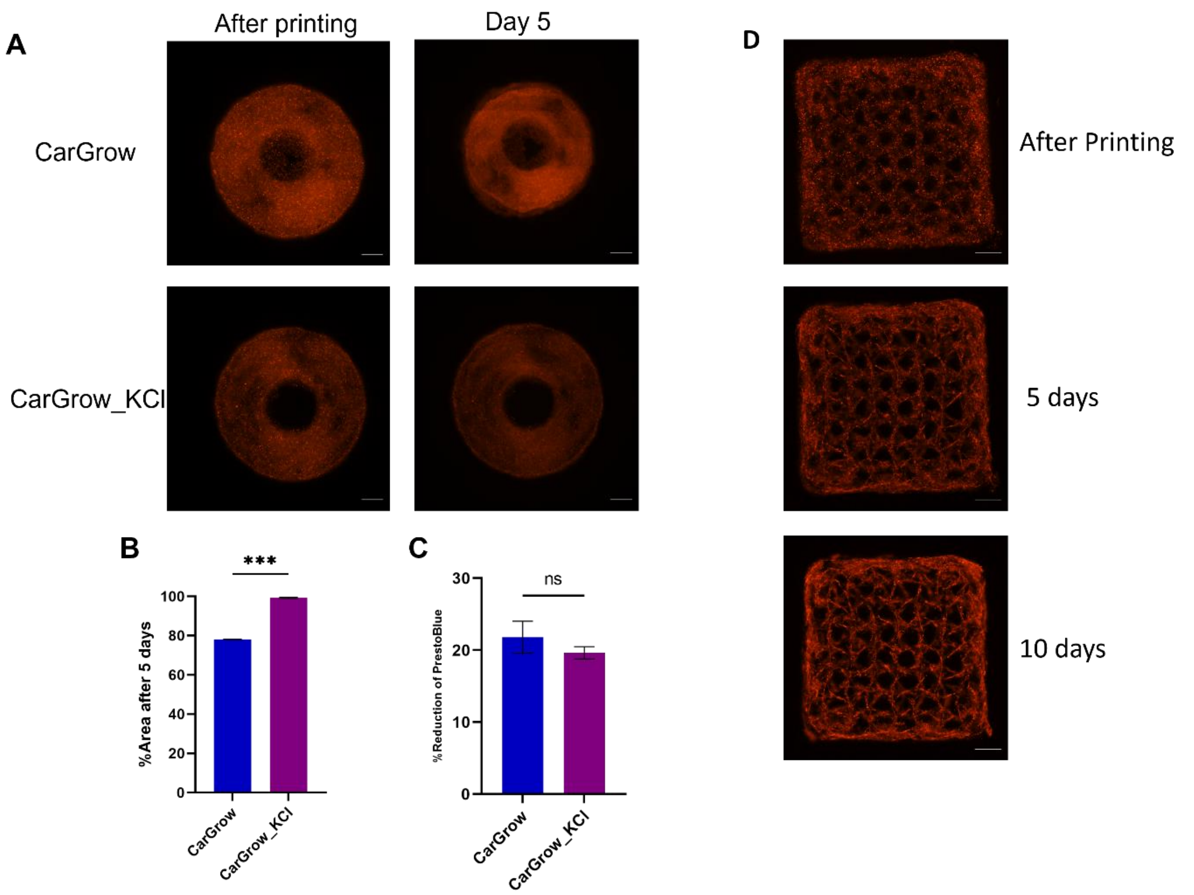

Figure S5. Suggested solutions to improve structure stability and reduced shrinkage constructs. (A) Fluorescence images of printed HNDF constructs using fibrin based bioink incubated within CarGrow. The constructs were cultured for five days within CarGrow and supplemented with normal medium or with 100mM KCl added to the medium (Scale bar: 1000µm). (B) Quantification of constructs area after five days compared to the area after printing. (C) Viability analysis of the printed constructs after five days using PrestoBlue technique. The results demonstrate significant structural stability due to the addition of 100mM KCl to the cell culture medium without significant difference in cell viability (Scale bar: 1000µm). (D) Fluorescence images of printed HNDF grid using fibrin based bioink incubated within CarGrow. The grid pattern was preserved during ten days of culture without significant deformation (Scale bar: 1000µm).

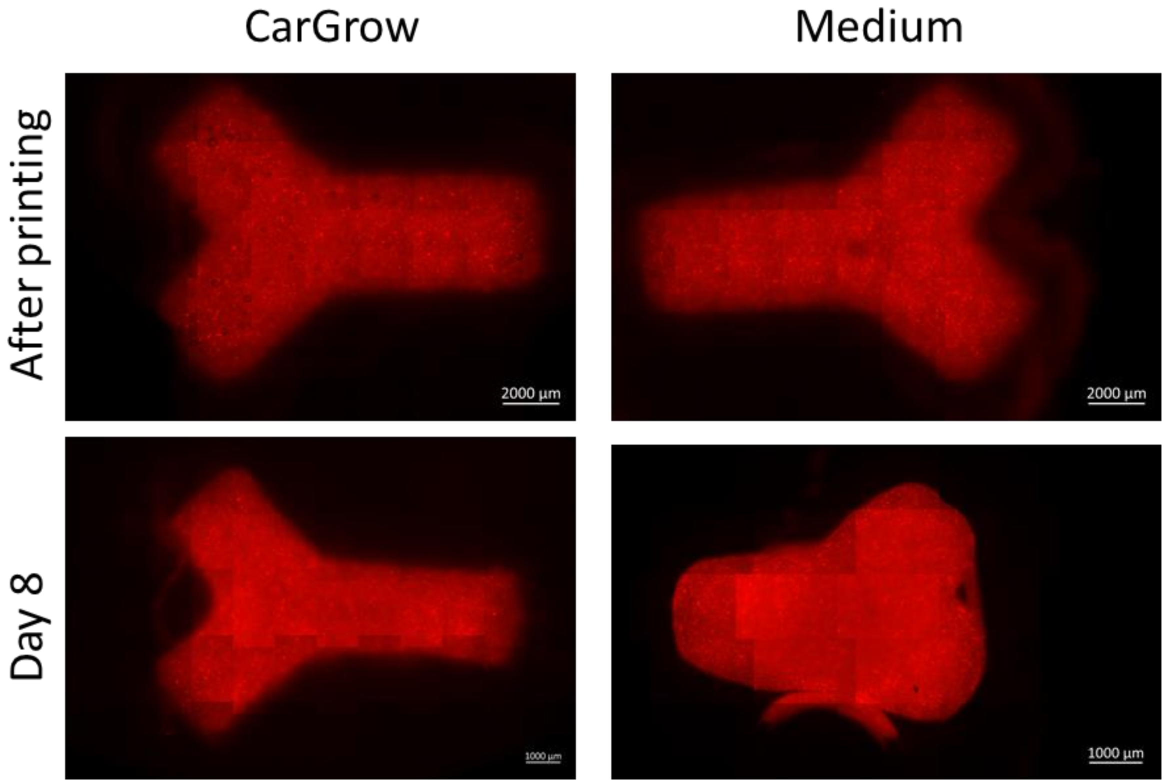

Figure S6. Cellular scaffold contraction and live cell imaging within CarGrow. Fluorescence images of printed HNDF constructs after printing and after eight days of culture. The printed constructs were cultured in a liquid medium or within CarGrow.

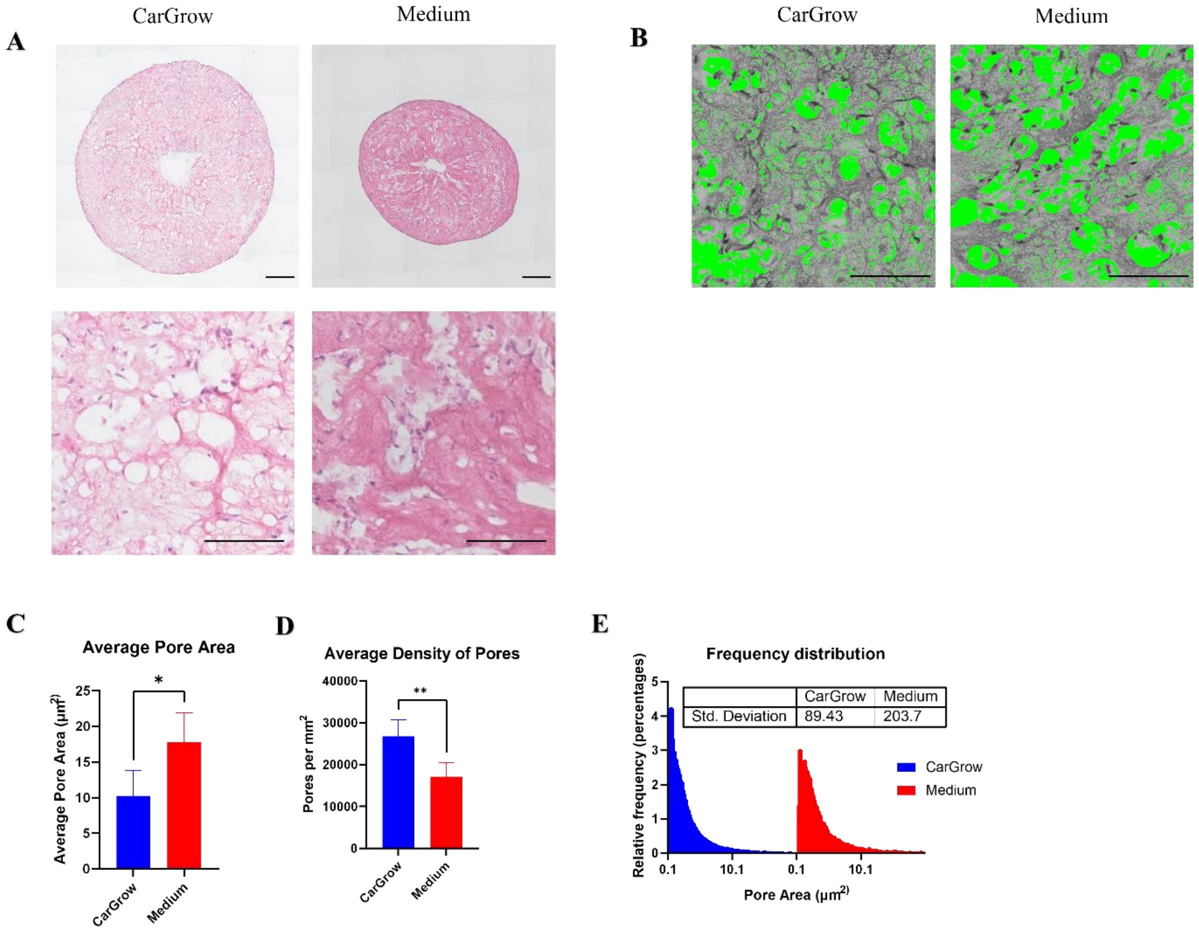

Figure S7. Quantification of constructs porosity after cultivation. (A) Hematoxylin and eosin stained cryo-sections of printed HNDF-laden fibrin constructs after seven days of cultivation in CarGrow or medium. (Scale bar: (top) 1 mm, (bottom) 100µm). (B) representative images depict the thresholding of greyscale images of the stained sections, showing the pore areas in green. (Scale bar: 100µm) (C) Quantification of the average pore areas (n = 5). (D) Quantification of the average density of pores (n = 5). (E) Frequency distribution of pore sizes showing a narrower distribution of pore sizes in constructs cultivated in CarGrow.

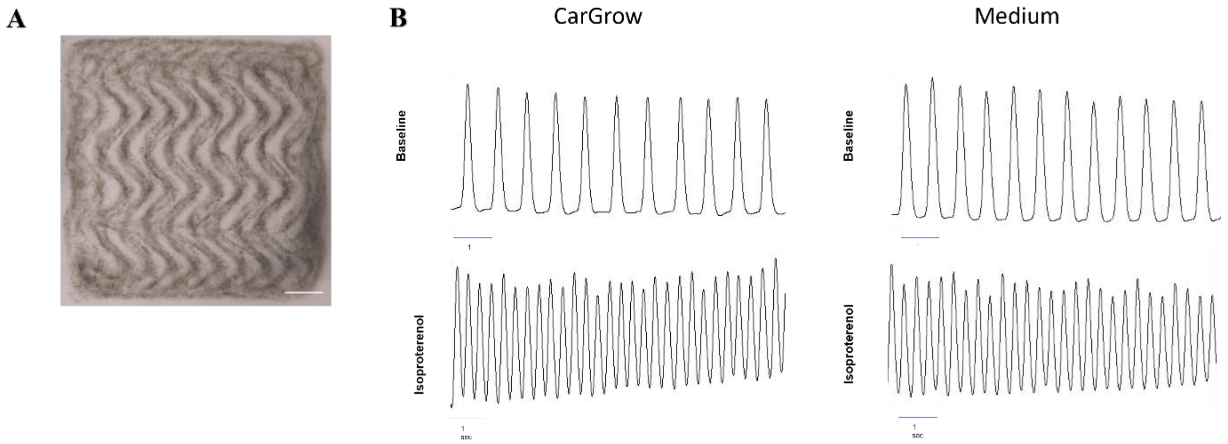

Figure S8. The functionality of printed cardiomyocytes-laden constructs. (A) Brightfield image of printed GcaMP-cardiomyocytes in a wave pattern (scale bar: 1 mm). (B) Representative graphs of the measured calcium transient signal of beating cardiomyocytes cultivated in CarGrow (right) and medium (left) before and after adding the drug (scale bar: 1 sec).

Movie S1. Perfusion of dye through printed contructs fabricated from alginate, gelMA, and fibrin.

Movie S2. Brightfield and fluorescent real-time imaging in CarGrow of GcaMP-cardiomyocytes spontaneously beating.
